# Among-individual differences in foraging modulate resource exploitation under perceived predation risk

**DOI:** 10.1007/s00442-020-04773-y

**Published:** 2020-11-03

**Authors:** Jana A. Eccard, Thilo Liesenjohann, Melanie Dammhahn

**Affiliations:** 1 Animal Ecology, Institute for Biochemistry and Biology, University of Potsdam, Maulbeerallee 1, 14469 Potsdam, Germany; 2 Berlin-Brandenburg Institute of Advanced Biodiversity Research (BBIB), Königin-Luise-Str. 2-4, 14195 Berlin, Germany

**Keywords:** Animal personality, Giving-up density, Intra-specific trait variation, Landscape of fear, Optimal foraging, Predation risk, Resource exploitation

## Abstract

Foraging is risky and involves balancing the benefits of resource acquisition with costs of predation. Optimal foraging theory predicts where, when and how long to forage in a given spatiotemporal distribution of risks and resources. However, significant variation in foraging behaviour and resource exploitation remain unexplained. Using single foragers in artificial landscapes of perceived risks and resources with diminishing returns, we aimed to test whether foraging behaviour and resource exploitation are adjusted to risk level, vary with risk during different components of foraging, and (co)vary among individuals. We quantified foraging behaviour and resource exploitation for 21 common voles (*Microtus arvalis*). By manipulating ground cover, we created simple landscapes of two food patches varying in perceived risk during feeding in a patch and/or while travelling between patches. Foraging of individuals was variable and adjusted to risk level and type. High risk during feeding reduced feeding duration and food consumption more strongly than risk while travelling. Risk during travelling modified the risk effects of feeding for changes between patches and resulting evenness of resource exploitation. Across risk conditions individuals differed consistently in when and how long they exploited resources and exposed themselves to risk. These among-individual differences in foraging behaviour were associated with consistent patterns of resource exploitation. Thus, different strategies in foraging-under-risk ultimately lead to unequal payoffs and might affect lower trophic levels in food webs. Inter-individual differences in foraging behaviour, i.e. foraging personalities, are an integral part of foraging behaviour and need to be fully integrated into optimal foraging theory.

**Electronic supplementary material:**

The online version of this article (10.1007/s00442-020-04773-y) contains supplementary material, which is available to authorized users.

## Introduction

A fundamental problem governing animal behaviour is to balance the benefits of foraging with the costs of becoming food for others (Krebs and Davies 1978). One singular erroneous assessment of predation risk, i.e. the likelihood of falling prey to a predator’s attack, may have fatal consequences for individual fitness. Therefore, natural selection has favoured adaptations of activity, foraging, space use, and sociality to minimise predation risk (Caro 2005). Moreover, predation risk also exerts a strong influence on behavioural variation over ecological time scales (Lima and Dill 1990; Lima 1998). Predators can affect the fitness of their prey either directly by killing it, or indirectly by scaring it. The changes in prey morphology, physiology, life-history and behaviour in reaction to the mere presence of a predator are collectively referred to as non-consumptive, non-lethal, or indirect predation effects (Lima 1998). These indirect effects are as or even more important in predator–prey interaction than direct predation effects (meta-analyses: Luttberg and Kerby 2005; Preisser et al. 2005). Indirect predation effects rely on perceived predation risk, i.e. the individual’s assessment of the likelihood of a predator's attack, which is based on experiences and imperfect knowledge of the environment (Bouskila and Blumstein 1992; Hill and Dunbar 1998). Perceived predation risk affects behavioural adjustments aimed at reducing immediate individual risk (Lima 1998) and involve energetic trade-offs and, thus, affect prey fitness indirectly. One of the best described and studied examples is the trade-off between balancing foraging gain and predation risk (Lima and Dill 1990; Lima 1998).

Perceived predation risk varies in space and time creating a landscape of fear (Brown et al. 1999; Laundré et al. 2001, 2014; Brown and Kotler 2004). Perceived predation risk is inversely proportional to time spent in a food patch, which is inversely proportional to the food density that a forager leaves in a patch when giving up foraging activities. This measure of giving-up density (GUD, Brown 1988) of food in artificial food patches, thus, provides an indirect method to quantify landscapes of fear and allows valuable insights into foraging under risk in a large number of small ground-dwelling mammals (summarised in Verdolin 2006; Bedoya-Perez et al. 2013). Small mammals decrease their foraging effort in habitats with increased perceived predation risk and habitat cues elicit clearer reactions than odour and live predators (Verdolin 2006). However, 20% of GUD-based studies included in the meta-analysis of Verdolin (2006) did not find support for a trade-off between energy gain and risk avoidance.

The large majority of studies addressing foraging and antipredatory behaviour under risk focused on heterogeneous landscapes of fear, for example in desert ecosystems with a choice of differently dangerous microhabitats (e.g. Kotler and Brown 1999; Dall et al. 2000). Risk-uniform landscapes have received very little attention, albeit—from a forager’s perspective—they are not uncommon (but see Eccard and Liesenjohann 2008; Eccard et al. 2008; Liesenjohann and Eccard 2008; Menezes et al. 2014). The available studies revealed that under uniform risk foragers may exploit a few patches only, particularly under high risk (Eccard and Liesenjohann 2008). Because changing between patches is costly in a uniform landscape of high risk, foragers accept small returns locally and thereby generate landscape-level variation in GUDs. Second, the patch model and GUD-based studies have mainly focused on perceived risk during foraging in a patch but changing between patches through a matrix can also be risky. The marginal value theorem (Charnov 1976) predicts that the costs (time spend) travelling between patches in a given environment determine the time in a patch, and, hence, GUD (Brown 1988). Since the movement of foragers is often unknown in GUD studies, we know very little about how other types of costs, e.g. predation risk during travelling, modify GUDs in a patch. Third, intrinsic individual differences in behaviour, i.e. animal personality (Réale et al. 2007), might contribute to unexplained variation in risk-taking during foraging (e.g. *Microcebus murinus*: Dammhahn and Almeling 2012; *Tringa totanus*: Couchoux and Cresswell 2012), although hitherto only few studies could estimate the magnitude of this effect (e.g. *Trichosurus vulpecula*: Mella et al. 2015). Inter-individual differences in foraging behaviour may appear to produce suboptimal foraging outcomes if considered isolated. However, if individuals differ in state (e.g. morphology, physiology), individual variation in foraging under risk could reflect adaptive solutions of balancing costs and benefits in a state-dependent manner (Houston and McNamara 1999). Moreover, these among-individual differences in risk-taking may be adaptively integrated in an extended pace-of-life syndrome (POLS) with other behavioural, physiological and life-history traits (e.g. Dammhahn et al. 2018). Thus, to better understand foraging decisions under risk and their cascading effects in landscapes of fear, we need to decipher landscape-level and individual-level contributions to variation in GUDs.

Therefore, we aimed to test (1) whether foraging of rodents in artificial landscapes of uniform risks and resources is adjusted to perceived risk level and type, (2) whether aspects of foraging behaviour and remaining resource landscapes vary with perceived risk during different components of foraging (dissecting travelling between patches and time in the patch), (3) whether individuals differ consistently in aspects of foraging under perceived predation risk, and (4) whether these potential among-individual differences in foraging behaviour are associated with individual patterns of landscape-wide resource exploitation. We used a widely distributed, ground-dwelling rodent, the common vole (*Microtus arvalis*) for our study because this species is representative for a typical prey species preyed upon by a variety of avian and mammalian predators (Stein 1958; Halle 1988, 1993). Previous studies in this species demonstrated that predation risk exerts a strong effect on the amount of resources remaining after exploitation (e.g. Jacob and Brown 2000). Furthermore, among-individual differences along the shy-bold and active-inactive axes are consistent across context and over time (Eccard and Herde 2013; Herde and Eccard 2013).

To test our hypotheses, we created simple artificial landscapes of perceived risks and resources for single foragers, and quantified aspects of foraging behaviour and landscape-wide patterns of resource exploitation. By manipulating ground cover, which ground-dwelling small mammals use as their proxy of predation risk (e.g. Brown 1988; Jacob and Brown 2000), we created foraging landscapes with low and high perceived risk. To tease apart the effects of various components of foraging under risk, we used a full factorial design and manipulated perceived predation risk while feeding inside a patch and while travelling between food patches. Specifically, we tested the following hypotheses and predictions: (1) perceived predation risk affects foraging behaviour, such as latency to resume feeding, activity, or changes among food patches, and food resource exploitation, such as food left in a patch (GUD) and evenness of GUDs among patches. In theory, an optimal forager should leave a patch when the return rate falls below the average of the habitat (Charnov 1976); while in a 2-patch set-up, the return rate of one patch should not fall below the other. A Bayesian forager would have to forage in both patches, compare and adjust, ultimately converging GUDs. (2) Perceived risk during travelling modifies not only foraging behaviour but also the remaining resource landscape. The costs of travelling between patches in a given environment influence the time in a patch (Charnov 1976). Thus, we predicted that (i) changes between food patches are more frequent when travelling is safe as compared to risky, (ii) under a given risk during feeding, minimal GUD is higher when travelling is safe as compared to risky, as changes to another food patch are more likely, and (iii) that foragers exploit the landscape more evenly if travelling is safe as compared to risky. (3) Foraging behaviour in landscapes of risks and resources varies among individuals. We expected aspects of foraging behaviour to be repeatable within individuals across different artificial landscapes of perceived risk. (4) Consequently, remaining resource landscapes vary among individuals so that also aspects of resource exploitation are repeatable within individuals across different artificial landscapes of perceived risk. (5) Among-individual differences in foraging behaviour and resource exploitation are functionally integrated. We expected negative among-individual correlations between foraging duration and minimal GUD (which indicates the longest, local risk-taking). Further, we expected positive among-individual correlations between foraging duration and absolute amount of food consumed as well as between patch changes and evenness of resource exploitation. (6) Individuals differ in foraging efficiency, i.e. consistently under- or overperform in exploitation, because they consistently vary in risk-taking. We expected individual deviations from population-level harvest curves to be repeatable across risk treatments.

## Methods

### Experimental subjects

We captured 24 (12 males; 12 females) common voles (*Microtus arvalis*) on the premises of the University of Bielefeld. Upon capture, subjects were transferred to single cages with hay, water, and rabbit food pellets ad libitum for 2–4 months prior to the experiment, kept in an outside aviary. All animals were adult and sexually active at capture. There was no difference in body mass between males and females (mean ± SD body mass: 22.9 ± 4.2 g, *t* test: *t* = 1.21, *df* = 22, *p* = 0.24).

### Experimental design

**Fig. 1:**
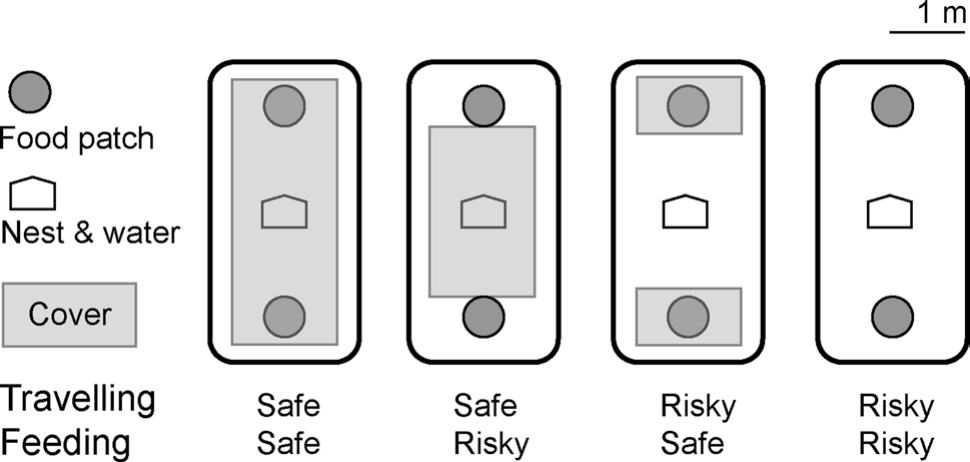
Common voles (*Microtus arvalis*) were placed in arenas with a nest and a water source in the middle (house symbol) and two food patches with diminishing returns (circles), placed at opposite sides of the arena. The presence or absence of ground cover (mesh wire of 1 cm × 1 cm, grey squares) at 2 cm height from the ground served to manipulate perceived predation risk. In a fully crossed factorial experimental design, we covered either food patches (feeding safe or risky) or the ground between food patches (travelling safe or risky)

Each animal was released singly into one of eight experimental arenas of 4 m × 2 m, surrounded by metal walls of 30 cm height on a concrete floor in otherwise empty outdoor aviaries caged 2.5 m high, with solid roofs. Hence, study subjects could perceive the presence of a set of natural predators by sound and smell, but predators were prevented from entering the aviaries. Each arena contained a central nest box and two food patches of 30 cm × 20 cm × 5 cm at either end of the arena (Fig. 1), each containing 2 l of fine sand into which we mixed 1 g of millet (initial food density: 0.5 g millet/1 l sand). With time spend in food patch, GUDs followed diminishing returns functions (Fig. 5a). By covering the floor and the food patches with wire mesh (mesh size 1 cm × 1 cm) on 2 cm props, we created an environment of perceived safety from avian predation. Touching the mesh wire while moving under it gives voles a haptic perception of safety, but allowed us to film and observe individual behaviour from above (e.g. Eccard and Liesenjohann 2008). For the animals to become familiar to the resource landscape and the 24 h rhythm of resource renewal in the food patches, we allowed an initial habituation phase of three days before data collection. Voles are polyphasic with 5–8 independent foraging bouts per day. Thus, subjects had experienced the resource landscape for a total of 15–34 foraging bouts prior to data collection. During habituation, all animals lost weight (3–23% of weight loss compared to the beginning of habituation) indicating increased activity and reduced caloric uptake relative to the restricted cage space and ad libitum food availability experienced before the trial (Table ESM5). Body weight measurements before and after habituation were closely related (linear *R*^2^ = 0.91). We used the measurement at the end of habituation as an indicator of individual body condition for further analyses of foraging behaviour. For the experiment, we used a fully crossed factorial design and manipulated either the food patches so that foraging became safe (patches covered with wire mesh) or risky (patches open), and/or the ground between food patches (Fig. 1). We tested animals also in a fifth treatment condition, in which half of the arena including one food patch was covered with wire mesh; this risk-heterogeneous treatment condition was analysed and discussed in detail in Eccard and Liesenjohann (2014), but was not used here.

We changed risk condition every 24 h. To avoid carry-over effects from the previously experienced risk condition, we allowed voles to habituate for 18 h after each change, before we observed their behaviour during a 6-h-long observation period (Liesenjohann and Eccard 2008) in the evening. At the start and at the end of each 6-h trial, we provided voles with two food patches containing 1 g millet seeds in 2 l of sand. To measure remaining resource landscapes after the observation period, food patches were sieved and the remaining millet seeds were dried and weighed to the next 0.01 g. GUD of food are expressed as millet (g) per litre sand. During each experimental trial we video-recorded all subjects using infrared cameras.

Each animal was subjected to each of the five treatment conditions in a pseudo-random order. In the first group of eight parallel tested animals, two did not start foraging and were removed from the experiment during habituation. Two animals were not foraging in some of the four treatment conditions, rendering gaps it the data. This behaviour may be due to risk avoidance but also to the polyphasic activity pattern of common voles (Halle and Lehmann 1987). To avoid such gaps in the second and third groups, we continued the experiment for the three animals with a missing treatment for 1–2 days, respectively, until we had obtained foraging data for all five treatments, meanwhile keeping the rhythm of 18 h habituation and 6 h observations between trials. In one arena (male vole) we had frequent camera failures and could not analyse behavioural data. Overall, GUD data of 82 experimental trials of 21 individuals, and 79 videos of 21 animals could be collected (raw data Table ESM3 and ESM4). Animals were returned to home cages directly after the experiment, and were released back to the wild at the individual point of capture.

We quantified the following behavioural variables from video analysis: cumulative duration spent active (i.e. full body without tail outside of nest), number and length of activity bouts (defined as continuous activity outside of nest), number of food patch changes, duration spent feeding (i.e. full body without tail in food patch), and latency to commence activity after disturbance (i.e. emergence from nest) starting the observation period with fresh food patches. Separate activity bouts were counted if inactivity (during which the subjects stayed hidden in its nest) was longer than 30 min. To quantify exploitation across the resource landscape, we measured absolute amount of food consumed as difference between provided and remaining food, highest local exploitation as the lower of the two GUDs, and evenness of exploitation at the end of each experimental trail. Evenness (E) ranged between 0 (very uneven) and 1 (very even) and was calculated as:

$$E = 1-((\text{food [g] left in patch 1}-\text{food [g]}\,\text{left in patch 2})/\text{absolute food consumed [g]}).$$

### Statistical analyses

To test whether common voles adjust aspects of foraging behaviour to the spatial distribution of perceived risk, we used (generalised) linear mixed-effects models (G/LMMs) run with the R packages *lme4* and *lmerTest* (Bates et al. 2015). For each dependent variable, we modelled the underlying error structure of the data via the corresponding distribution family and link function; continuous variables (latency to emerge, duration active, duration feeding) were log-transformed. We included risk during feeding (high or low) and during travelling (high or low) and their interaction as fixed effects. We controlled for repeated measures of individuals by including a random effect individual, specified as random intercept. To control for potential effects of body mass differences at the beginning of the experiment, order of treatments, i.e. habituation to the set-up, and differences between the sexes, we included all of these control variables in initial models as fixed effect. Since these variables were not part of our hypotheses, we used step-wise backward model selection to remove the interaction and to excluded control variables if they did not improve model fit, assessed by comparing nested complex and simpler models using LR-tests (Zuur et al. 2009). If the interaction between feeding and travelling risk improved model fit, we ran post-hoc analyses for simple effects of one treatment (e.g. feeding risk) within one level of the respective other treatment (e.g. travelling risk) and vice versa. We assessed model fit based on residual distribution using qqplots. We estimated the proportion of explained variance by the fixed factors alone (marginal *R*^2^) and by fixed and random factors together (conditional *R*^2^) using the R package *MuMIn* (Nakagawa and Schielzeth 2013); for GLMMs we report lognormal *R*^2^.

To test whether aspects of foraging behaviour and of resulting exploitation landscapes are repeatable across risk landscapes, we estimated repeatability of behavioural variables across experimental trials using the R package *rptR* (Nakagawa and Schielzeth 2010; Stoffel et al. 2017). We adjusted repeatability estimates for treatment condition. For each dependent variable, we specified the underlying error distribution. We used 1000 simulations to estimate confidence intervals and 1000 permutations to estimate *p*-values.

Based on bivariate Bayesian mixed-effects models, run with the R package *MCMCglmm* (Hadfield 2010) and following procedures described in Dingemanse and Dochtermann (2013) and Houslay and Wilson (2017), we estimated among-individual correlations between pairs of dependent variables (feeding duration and minimal GUD, feeding duration and absolute amount of food consumed, and frequency of tray changes and evenness of exploitation, respectively). Due to small sample size, we added only the risk during feeding (high or low) as a fixed effect because this risk modality had larger effects on all response variables (see results below). All models included individual as a random effect. We set slightly informative priors by dividing the total phenotypic variance of the dependent variables by the number of random effects in the model and set a low degree of belief (nu = 1) because we do not have much information regarding the posterior distribution of the data (Hadfield 2010). Model results were robust against different priors (flat uninformative; results not shown). Error structures of the data were modelled via the underlying distribution families of the response variables. We used 220,000 iterations, a thinning interval of 100 and a burnin of 20,000, which resulted in low temporal autocorrelation between estimates of subsequent models. Based on the posterior distributions, we extracted among-individual and within-individual correlations between pairs of response variables and their credibility intervals; correlations were interpreted significant if the credibility intervals did not include zero (Houslay and Wilson 2017). Finally, to test for among-individual differences in efficiency of resource exploitation (over- and underperformance), we estimated a population-level harvesting curve, using cumulative foraging duration in each of the two presented patches, i.e. patch residence time, as the predictor variable and food consumed of the respective patch as the response variable. An exponential function was fitted to the complete data using the R function *nls*. In prior analyses, we fitted functions to data sets separated by treatments but the functions were very similar (not shown). Subsequently, we estimated residuals to the population harvesting curve and obtained repeated samples of individual deviation from population-level patch exploitation. We estimated repeatability of these individual deviations in exploitation adjusted for risk treatment, body mass, order of treatment presentation, and food consumed. All statistical analyses were performed with the program R (Version 3.3.0; R Core Team 2016) using the specified packages. The level of significance was set at *α* < 0.05.

### Ethical note

For this study, we documented the natural foraging behaviour of animals in a controlled environment. We did not consider the study as an animal experiment under the German Animal Protection Law (TierSchG *§*7(1)1) because it inflicted no pain, suffering or defects to the subjects. There was no physical treatment, aggressive interaction, or risk of injury for the animals. Capture, animal care and housing complied with institutional guidelines, permissions were given by the following authorities: Landesamt für Natur-, Umwelt- und Verbraucherschutz North-Rhine Westphalia (9.93.2.10.42.07.069 behavioural experiments, 360.12.06.01.3 capture of rodents from the wild), Gesundheits-, Veterinär- und Lebensmittelüberwachungsamt Bielefeld (530.42, keeping and breeding of wild captured voles).

## Results

### Foraging behaviour varied among risk treatments

Following a disturbance (i.e. refilling of foraging stations at the beginning of a 6-h observation period) animals commenced activity after latencies of 90 ± 75 min (mean ± SD; range 0–4 h 33 min). During an observation period, subjects were active for 36 ± 37 min (12 s–3 h 30 min) and spent 18 ± 24 min (10 s–2 h 27 min) feeding at the food patches. Foraging activity was spread over 2.5 ± 1.3 bouts (0–6). Animals changed 5.3 ± 5.2 times between foraging stations (0–25), equalling 11.8 ± 10.9 changes per hour feeding time. In four experimental trials, animals foraged in both food patches but never changed directly between patches because they targeted only one patch per activity bout and returned to the nest in-between foraging bouts.

**Fig. 2:**
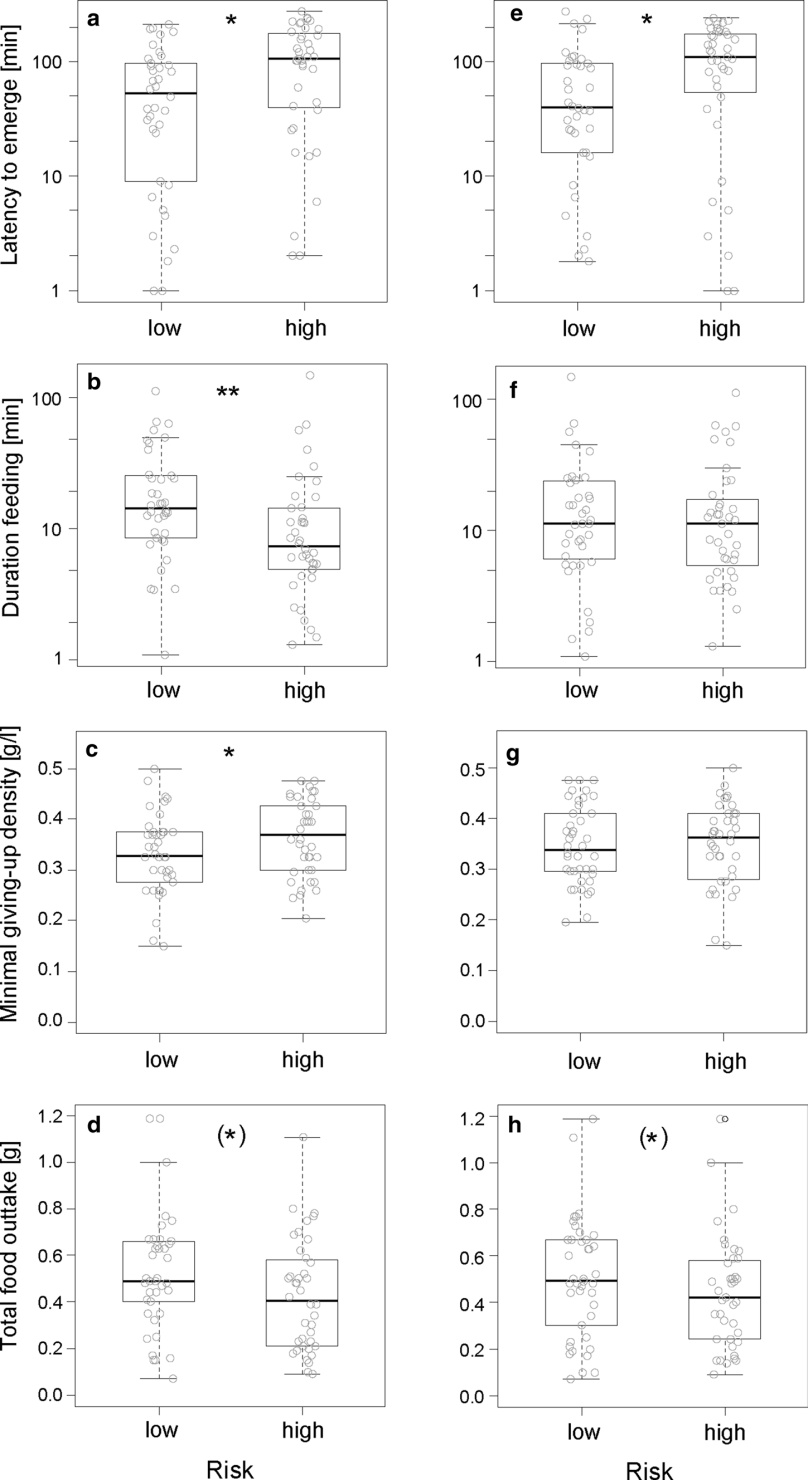
Foraging behaviour (latency to emerge after disturbance, duration feeding) and resource exploitation (minimal GUD, absolute amount of food consumed) of common voles (*Microtus arvalis*) in artificial resource landscapes of high and low risk during foraging (**a**–**d**: left column) and travelling (**e**–**h**: right column). Shown are median (line), inter-quartile range (boxes), min–max range (whiskers) and raw data (dots). Significant differences are indicated as * for *p* < 0.05 and ** for *p* < 0.001 and as (*) for *p* < 0.1 for a statistical trend

**Fig. 3:**
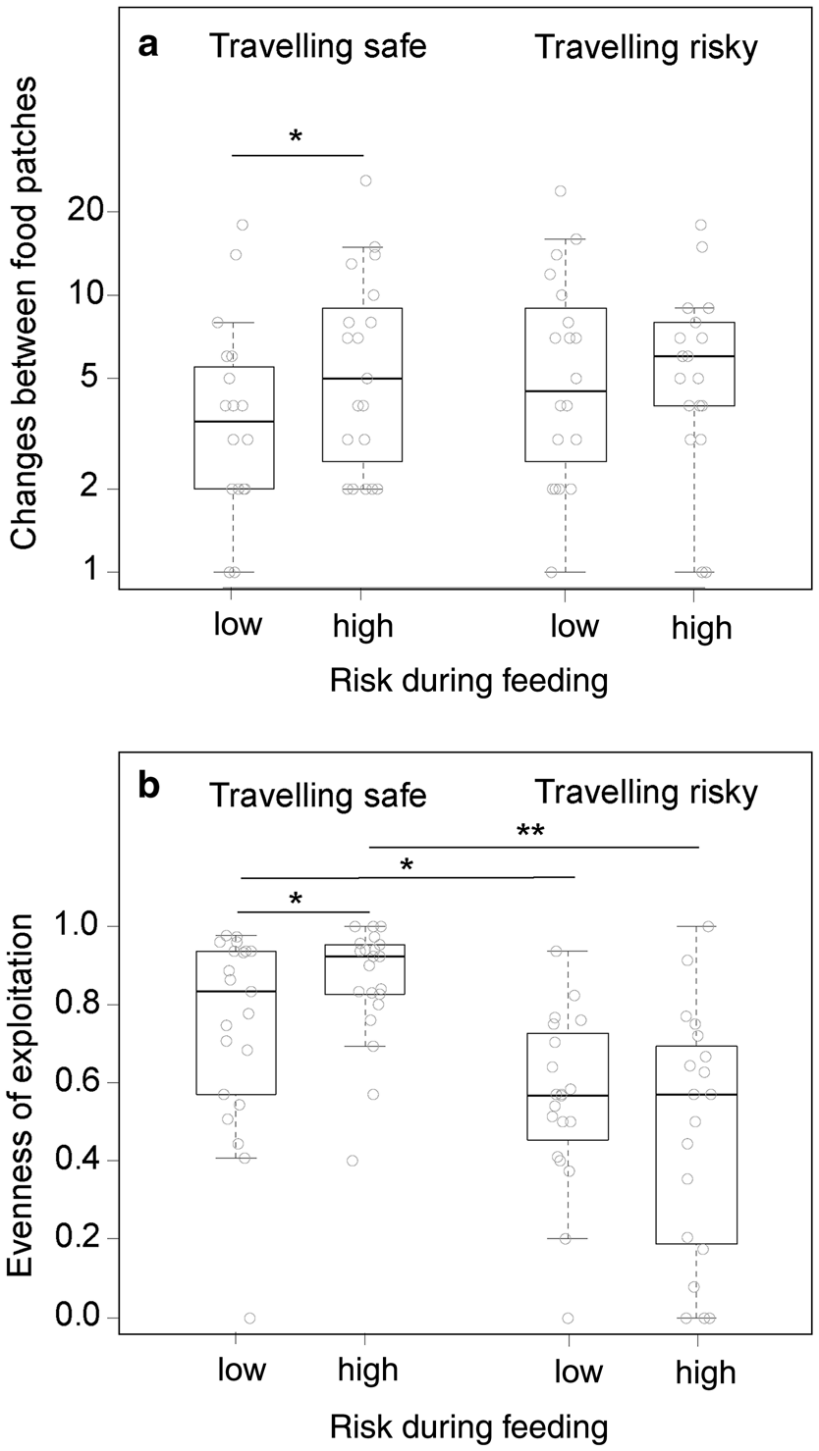
Common voles (*Microtus arvalis*) foraging in artificial resource landscapes with high or low risk during travelling and feeding adjusted the number of changes between two food patches and the evenness of exploiting the two food patches to a combination of the two risk types. Shown are median (line), inter-quartile range (boxes), min–max range (whiskers) and raw data (dots). Significant differences are indicated as * for *p* < 0.05 and ** for *p* < 0.001

Foraging behaviour varied between risk treatments. Animals took longer (latency) to emerge from shelter when risk during feeding was high compared to low (Table 1; Fig. 2). Similarly, latency was longer when travelling was risky compared to safe (Table 1; Fig. 2). There was no interaction between the two risk modalities (LMM: *χ*^2^ = 1.03, *p* = 0.310). The order of treatments (i.e. habituation to the arenas) did not affect latency to emerge and males tended to emerge later than females (Table 1). There was a tendency for an interaction between travelling risk and feeding risk for the duration active (Table 1), but none of the post-hoc tests revealed a significant effect of one risk modality within the other (all post-hoc tests: *p* > 0.07). Animals decreased activity over the course of the experiment (order effect) and the heavier an individual had been initially (before testing), the shorter its activity duration (sum of activity bout lengths). The duration feeding was lower when feeding was risky as compared to safe (Table 1; Fig. 2). Risk during travelling had no influence on feeding duration and the interaction between risk modalities was non-significant (LMM: *χ*^2^ = 0.12, *p* = 0.734). Subjects spent less time feeding over the course of the experiment and feeding time scaled negatively with initial body mass (Table 1). The number of tray changes was explained by an interaction between the two risk modalities (Table1; Fig. 3). Specifically, only if travelling was safe tray changes occurred more often when feeding was risky as compared to safe (post-hoc test: *β* ± SE = 0.70 ± 0.17, *p* < 0.001). If travelling was risky, tray changes were independent of feeding risk (post-hoc test: *β* ± SE = − 0.002 ± 0.144, *p* = 0.987). Moreover, for neither of the two feeding risk treatments did tray changes differ between safe and risky travelling (post-hoc test for low feeding risk: *β* ± SE = 0.30 ± 0.16, *p* = 0.058; post-hoc test for high feeding risk: *β* ± SE = − 0.15 ± 0.14, *p* = 0.261).

**Table 1:** Results of (generalised) linear mixed-effects models of variables quantifying foraging behaviour of common voles (*Microtus arvalis*) in experimental resource landscapes varying in risk distribution

Variable	Latency to emerge (min)		Duration active (min)		Duration feeding (min)		Patch changes	
	*β* ± SE	*p*	*β* ± SE	*p*	*β* ± SE	*p*	*β* ± SE	*p*
Intercept	2.66 ± 0.40		3.62 ± 0.28		7.31 ± 1.12		1.41 ± 0.21	
Body mass			− **0.11 ± 0.04**	**0.009**	− **0.15 ± 0.04**	**< 0.001**		
Sex	0.80 ± 0.45	0.077						
Order			− **0.20 ± 0.06**	**< 0.001**	− **0.24 ± 0.06**	**< 0.001**	− **0.12 ± 0.04**	**0.001**
Risk feeding	**0.79 ± 0.26**	**0.002**	0.25 ± 0.22	0.781	− **0.48 ± 0.15**	**0.001**	**0.60 ± 0.15**	**< 0.001**
Risk travelling	**0.66 ± 0.25**	**0.009**	0.36 ± 0.23	0.701	− 0.13 ± 0.15	0.394	**0.41 ± 0.15**	**0.006**
Risk feeding × risk travelling			− 0.59 ± 0.31	0.059			− **0.64 ± 0.20**	**0.001**
*R*^2^_marginal_		0.17		0.24		0.38		0.11
*R*^2^_conditional_		0.47		0.53		0.63		0.73
Random effect variance		0.37		0.39		0.39		0.39

Shown are effects (*β*) and their standard error (SE) as well as *p* values from model comparisons based on LR-tests, proportion of variance explained by the random effect individual, proportion of variance explained by the fixed effects (*R*^2^_marginal_) and by fixed and random effects (*R*^2^_conditional_). Significant effects are marked in bold. Non-significant control variables and interactions were removed from models (threshold < 0.1), indicated by empty cells. The reference level is “low risk” for both categorical risk modalities and female for sex. Order refers to the sequence of treatment presentations from 1 to 5

### Exploitation landscapes varied with risk treatments

Food consumption per animal was 0.47 ± 0.25 g millet over 6 h (0.07–1.19 g millet), resulting in a remaining resource landscape with GUDs of 0.35 ± 0.09 g millet/l sand (0.15–0.48 g/l). We found a difference of remaining resources between the two food patches of 0.07 ± 0.06 g/l (0.00–0.24 g/l), translating into an evenness of depletion among the two patches of on average 0.33 (range 0–1).

Remaining resource landscapes varied with risk treatments. Absolute food consumption tended to be lower if feeding or travelling was risky compared to safe (Table 2; Fig. 2), but there was no interaction between the two risk modalities (LMM: *χ*^2^ = 0.07, *p* = 0.395). Food consumption decreased over the course of the experiment (order effect). The minimal GUD was higher when foraging risk was high, but did not vary with travelling risk (Table 2; Fig. 2). The interaction between risk modalities did not explain variation in minimal GUD. The evenness of food exploitation was explained by an interaction of risk during travelling and risk during feeding (Table 2; Fig. 3). Specifically, evenness was lower in conditions under which travelling was risky compared to safe, independent of the feeding risk condition (post-hoc test for high risk during feeding: *β* ± SE = − 0.48 ± 0.11, *p* < 0.001; post-hoc test for low risk during feeding: *β* ± SE = − 0.21 ± 0.09, *p* = 0.012). If travelling was risky, evenness was independent of feeding risk (post-hoc test: *β* ± SE = − 0.11 ± 0.11, *p* = 0.302). However, if travelling was safe, evenness was higher when feeding was risky as compared to safe (*β* ± SE = 0.19 ± 0.08, *p* = 0.015).

**Table 2:** Results of (generalised) linear mixed-effects models of variables quantifying remaining resource landscapes of common voles (*Microtus arvalis*) foraging under different risk treatments

Variable	Food consumption (g)		Minimal GUD (g/l)		Evenness	
	*β* ± SE	*p*	*β* ± SE	*p*	*β* ± SE	*p*
Intercept	1.12 ± 0.25		0.33 ± 0.01		1.20 ± 0.10	
Body mass	− 0.02 ± 0.01	0.069				
Order	− **0.04 ± 0.02**	**0.027**			− 0.04 ± 0.03	0.072
Risk feeding	− 0.09 ± 0.05	0.061	**0.03 ± 0.02**	**0.033**	0.18 ± 0.09	0.494
Risk travelling	− 0.09 ± 0.05	0.081	0.00 ± 0.02	0.904	− **0.21 ± 0.09**	**< 0.001**
Risk feeding × risk travelling					− **0.29 ± 0.13**	**0.030**
*R*^2^_marginal_		0.16		0.04		0.29
*R*^2^_conditional_		0.25		0.25		0.29
Random-effect variance		0.11		0.22		0.01

Shown are effects (*β*) and their standard error (SE) as well as *p* values from model comparisons based on LR-tests, proportion of variance explained by the random-effect individual, proportion of variance explained by the fixed effects (*R*^2^_marginal_) and by fixed and random effects (*R*^2^_conditional_). Significant effects are marked in bold. Non-significant control variables and interactions were removed from models, indicated by empty cells. The reference level is “low risk” for both categorical risk modalities. Order refers to the sequence of treatment presentations from 1 to 5

### Among-individual differences in behaviour and exploitation across risk treatments

**Fig. 4:**
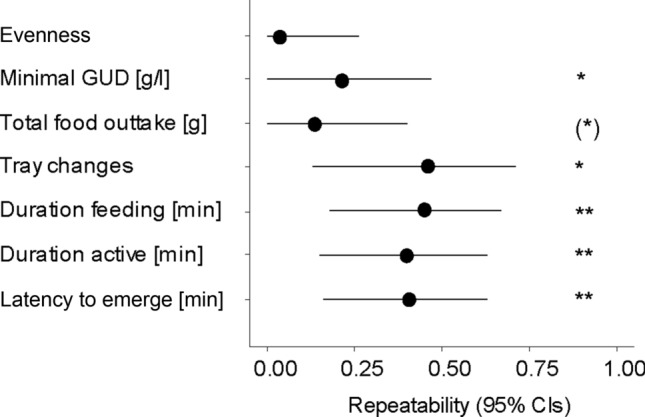
Repeatability estimates of foraging behaviour and remaining resource landscapes of common voles (*Microtus arvalis*) adjusted for the four different treatment conditions. Shown are repeatability estimates and their lower and upper 95% confidence intervals. Significant repeatability, as based on LR-tests, are indicated as * for *p* < 0.05, ** for *p* < 0.001, and as (*) for a statistical trend (*p* < 0.1)

**Fig. 5:**
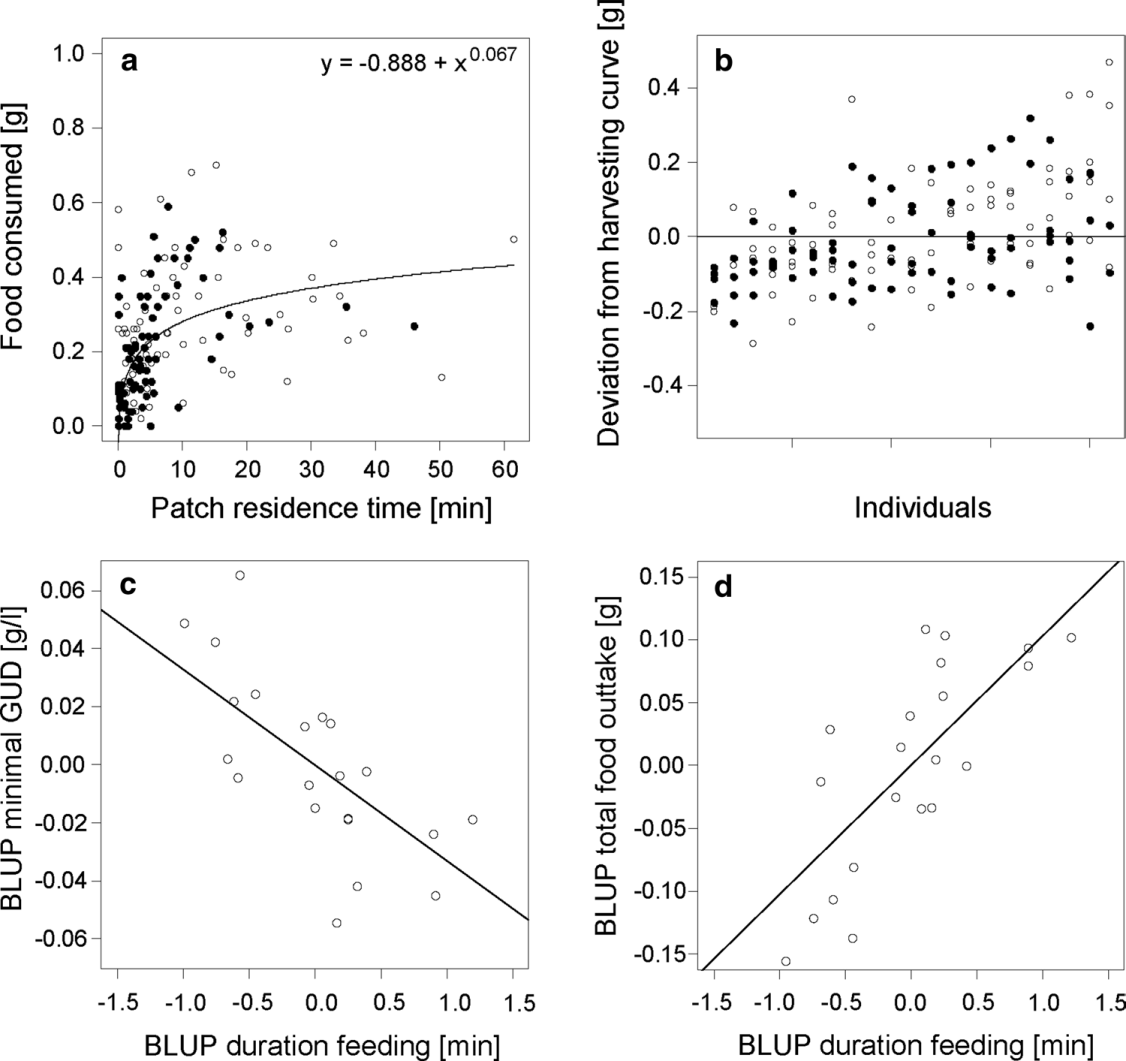
Harvesting of food patches by 21 common voles (*Microtus arvalis*): **a** population-level harvesting curve fitted to raw data of food consumed per cumulative patch residence time for risk landscapes of low feeding risk (open dots) and of high feeding risk (black dots); **b** individual performance residuals, i.e. deviations from the population harvesting curve, with individuals sorted by their mean residual value. **c**, **d** Inter-individual differences persist across different landscapes of risks and resources: among-individual correlations between foraging duration and lowest GUD (**c**) and absolute amount of food consumed (**d**). Shown are regression lines and BLUPs, i.e. individual random intercepts (one dot per individual), estimated—for illustrative purposes only—from bivariate Bayesian mixed-effects models (see “Methods” for details) across treatment conditions

All quantified aspects of foraging behaviour were highly repeatable across risk treatments (all *R*_adjusted_ > 0.40, all *p* < 0.001; Table ESM1; Fig. 4). In contrast, aspects of remaining resource landscapes were only marginally repeatable across risk treatments for minimal GUD (*R*_adjusted_ = 0.22, *p* = 0.017) or not repeatable (Table ESM1; Fig. 4). At the population level, harvesting at patches followed a function classical for depletable resources, with high returns for a short stay and diminishing returns for longer stays (Fig. 5a). Individuals were not equally efficient: they varied in deviation from this population harvesting function (Fig. 5b) and this deviation was repeatable across risk treatments (*R*_adjusted_ = 0.28, CI: 0.09, 0.47, *p* < 0.001).

In all models testing the effects of risk treatments on foraging behaviour, random and fixed effects together explained substantially more variation in the data than fixed effects alone (all *R*^2^_conditional_–*R*^2^_marginal_ > 0.29) and between-individual differences accounted for 37–39% of the variation, supporting the importance of variation among individuals as indicated by the repeatability estimates. In models testing the effects of risk treatments on remaining foraging landscapes, random effects added less explained variation (between 0 and 21%) and between-individual differences accounted for only 1–22% of the variation. At the between-individual level, duration feeding was negatively correlated with minimal GUD (*r* = − 0.53, CI: − 0.90, − 0.08, Fig. 5c) and positively correlated with absolute food consumption (*r* = 0.56, CI: 0.10, 0.91; Fig. 5d; within-individual correlations Table ESM2). The number of patch changes and evenness did not correlate at the between-individual level (*r* = 0.08, CI: − 0.44, 0.58).

## Discussion

Most animals have to balance foraging gain and predation risk, rendering this trade-off a fundamental influence on animal behaviour (Lima and Dill 1990; Lima 1998). Here, we manipulated such a trade-off by exposing individuals of a typical prey species, the common vole, to experimental landscapes of risks and resources. We found that individuals adjusted their foraging to perceived predation risk and expressed highly variable foraging behaviour resulting in variable remaining resource landscapes. Overall, risk during feeding in food patches was a stronger predictor of foraging behaviour and remaining resource landscapes than risk during travelling between two food patches. Risk during travelling modified the risk effects of feeding, which was evident for changes between food patches and the resulting evenness of the exploitation landscape. A significant part of variation in foraging under risk was due to consistent differences among individuals in when and how they exploited resources and exposed themselves to potential risk. These substantial among-individual differences in foraging behaviour correlated with individual differences in resource exploitation across risk treatments. Additionally, consistent under- or overexploitation of patches created further variation in individual payoffs. Below, we discuss these findings in more detail.

### Foraging behaviour and resource exploitation vary with perceived predation risk

As predicted, foraging behaviour of common voles in artificial landscapes of risks and resources was highly variable and was adjusted to risk level. If perceived predation risk was high, individuals entered the resource landscapes later, spent less time foraging and changed less often between patches, compared to lower perceived predation risk. As expected, higher levels of foraging behaviour resulted in higher levels of resource exploitation, which confirms that we indeed created a simple landscape of fear (Laundre et al. 2001). Overall, risk during feeding inside a patch was a better predictor of foraging behaviour and remaining resource landscapes than risk during travelling to and from the patch. Particularly, the highest local resource exploitation, i.e. the minimal GUD, in the landscape, was determined by risk during feeding only. Since GUD reflects the quitting harvest rate at which a forager leaves a patch, it is inversely proportional to the time spent feeding in a patch (Brown 1988). Thus, in safe conditions, foragers stayed longer, accepted diminishing returns, and quitted patches at lower GUDs. Consequently, absolute food consumption also tended to be higher in safer as compared to riskier conditions. These results are in line with many other experimental studies (meta-analysis: Verdolin 2006). Most of these studies only monitor the outcome of foraging decisions under risk. By directly observing foragers, we additionally demonstrated that individuals do adjust several aspects of foraging behaviour to risk conditions, confirming fundamental assumptions of the patch model (Brown 1988).

### Risk during travelling creates variation between patches

Foraging in a patchy landscape, a mobile forager has to evaluate the risk during feeding against the risk during travelling between patches. Travelling time between patches in a given landscape determines how long a forager should stay in a patch with diminishing returns (Charnov 1976). When travelling time between patches increases, the mean return value of the landscapes decreases and foragers should stay longer. Since perceived risk might represent an additional cost of travelling, the decision to quit a patch, i.e. GUD, should be determined not only by the risk in the patch but also by the risk of changing to a new patch. Here, we experimentally teased apart the risk during these two components of foraging and found that risk during travelling modified risk effects of feeding for some but not all aspects of foraging behaviour. In line with our prediction, foragers changed more often between unsafe food patches when travelling was safe. Consequently, foragers exploited a landscape more evenly if the risk during travelling was low. Notably, when both food patches were risky, foragers spreat their exploitation evenly between them. These results suggest that only if the costs of changing between food patches are low, foragers can adopt a marginal value strategy of optimal foraging. Thus, depending on the risk properties of a matrix between food patches, foragers will either equalise patch quality (marginal value theorem) or accept an increased heterogeneity between food patches. This variation in spatial distribution of individual foraging effort across a patchy landscape should have population-wide effects. Moreover, unequal exploitation of resource patches likely has cascading effects on the meta-population and community dynamics of the food species (e.g. McArthur et al. 2014). Surprisingly, increasing the risk during travelling did not affect minimal GUD in the landscape, although individuals tended to forage less (lower absolute food consumption) when travelling was risky compared to safe. Thus, in our small and uniform landscapes, missed opportunity costs during foraging appear insensitive to travelling risk (Eccard and Liesenjohann 2008).

### Individuals differ in foraging behaviour under perceived risk

In artificial landscapes of risks and resources, common voles showed consistent individual differences in several aspects of foraging behaviour. Nearly half of the total phenotypic variance of repeated measures of activity, foraging activity, latency to resume foraging after a disturbance, and of patch changes was due to differences among individuals. These repeatability values compare well to other species and behaviours (summarised in Bell et al. 2009). Notably, among-individual differences in foraging behaviour were not explained by key state variables affecting energetic needs, such as body mass and sex, for which we controlled in our models. Hence, even when exposed to very different environmental conditions, individual foraging behaviour was shaped to a large degree by intrinsic differences among individuals. These differences are not due to simple allometric scaling of energy requirements with body mass or coarse state differences between the sexes. To reduce interference with the subjects, we had not obtained body mass variation over the short experimental trials and, therefore, cannot test for within-individual variation of foraging behaviour with fine changes in body mass state. Overall, these results render the assumption of patch use models (but see Brown 1988) unlikely, that missed opportunity costs (MOC) and energetic cost (C) terms are constant for different individuals tested in the same conditions [e.g. differential asset protection (Clark 1994), different energy budgets (Stephens and Charnov 1982)]. In line with this argument, a significant proportion of studies (20%) included in a meta-analysis did not find support for a trade-off between energy gain and risk avoidance (Verdolin 2006). Intrinsic individual differences in behaviour contribute to this apparently suboptimal risk-taking during foraging [e.g. grey mouse lemurs: Dammhahn and Almeling 2012; brushtail possums: Mella et al. 2015, bank voles: Mazza et al. 2019]. Analysing movement data of far-ranging natural foraging trips of wandering albatrosses, Patrick et al. (2017) also discovered consistent individual differences in exploration–exploitation syndromes. In eastern grey kangaroos, females differed consistently in foraging and vigilance as well as in how they traded off foraging and antipredator behaviour (Favreau et al. 2014). Thus, among-individual differences in foraging behaviour under risk are widespread and future studies should address whether they are part of wider behavioural and pace-of-life syndromes (as suggested by McArthur et al. 2014; Toscano et al. 2016; Moran et al. 2017; Mazza et al. 2019). Theoretically, consistent differences in states (e.g. metabolic rate, residual reproductive value) will modify the balance between behaviours favouring foraging gain or risk avoidance (e.g. Rands et al. 2003; Luttbeg and Sih 2010, McElreath and Strimling 2006; Wolf et al. 2007; Houston 2010). These state-dependent models of adaptive consistent individual differences in behaviours (summarised in Dingemanse and Wolf 2010) provide testable predictions but we still lack empirical tests of state-dependent variation in foraging under risk which manipulated states repeatedly within individuals. Finally, more dynamic patch models, allowing systematic individual variation in all terms determining quitting harvest rate, i.e. energetic costs, predation risk and missed-opportunity costs (Brown 1988), will help to derive more nuanced predictions for variation of foraging under risk.

### Unequal foragers: functional integration of foraging behaviour and resource exploitation

Common vole individuals differed in how long they foraged and these individual differences were associated with consistent individual differences in resource exploitation patterns and in payoffs. Independent of the risk treatment, some individuals foraged longer and thereby gained more resources overall. In addition, individuals foraging for longer also had lower quitting harvest rates and, thus, appeared to perceive lower predation risk across all treatment conditions than individuals spending less time in food patches (Brown 1988; Brown and Kotler 2004). But why do some individuals have lower foraging costs and gain more resources than others? There are several non-mutually exclusive explanations.

Individuals might consistently vary in their perceptive abilities. Since all experimental subjects were healthy upon external inspection and did not show signs of sensory impairments, we regard individual variation in perception as unlikely. Alternatively, wild-caught common voles might have had different experiences of direct and indirect predation risk priming certain individuals to behave more cautious than others. Since our study species experiences high and ubiquitous predation risk (Stein 1958; Halle 1988, 1993) and all individuals were several weeks of age when captured, we assumed all individuals having had some experience with predation risk; further, the experimental set-up was new for all subjects. Therefore, we regard variation in previous experiences as unlikely to fully explain the observed large among-individual variation in risk-taking. Third, individuals might differ in state-dependent missed opportunity costs (MOCs), i.e. all fitness costs payed by not engaging into any other activity than foraging, such as, for example, territory defence, energy conservation, or residual reproductive value (asset protection, Clark 1994) or mate choice (Brown 1988). As argued by Eccard and Liesenjohann (2008), MOCs should be a strong determinant of local patch exploitation decisions in landscapes where predation risk is uniform among patches. All experimental subjects were mature males and females, with females non-gravid and non-lactating; thus variation between individuals in MOCs in an experimental setting deprived of many options of alternative fitness-increasing behaviour was greatly reduced, but age differences might significantly contribute to explaining variation in perceived risk between individuals under natural settings. Fourth, individuals might differ in the costs of foraging because they pay unequal energetic costs, as indicated by great differences in the relative weight loss during habituation. In contrast to a general assumption of the patch use model (Brown 1988), metabolic rate varies among individuals and is highly repeatable over time (meta-analysis: Nespolo and Franco 2007) and, hence, intrinsic among-individual differences in the idling costs of the organism should affect empirical measures of GUD. Basal metabolic rate of our study subjects was unknown, but we measured body mass of our study animals twice (before and after habituation), and controlled for body mass—an important determinant of metabolic rate—in our models.

Finally, variation in perceived risk might reflect intrinsic individual differences in risk-taking, as documented in fish (e.g. *Dicentrarchus labrax*: Killen et al. 2013), birds (e.g. *Parus major*: van Oers et al. 2004, Abbey-Lee et al. 2016, Abbey-Lee & Dingemanse 2019; *Tringa totanus*: Couchoux and Cresswell 2012), and mammals (e.g. *Microcebus murinus*: Dammhahn and Almeling 2012; *Trichosurus vulpecula*: Mella et al. 2015), and ought to be linked to individual variation along a risk–reward trade-off (Réale et al. 2007; Sih and DelGiudice 2012). Thus, more risk-taking individuals are expected to trade off safety over resource gain; whereas risk-avoiding individuals favour safety and accept lower returns. In our experiment, individuals that consistently exposed themselves for longer to potential risk gained more food overall. Similarly, fast behavioural types of bank voles (*Myodes glareolus*) exploited high-risk food patches to lower GUDs and gained higher payoffs, as compared to their slow conspecifics (Mazza et al. 2019). While in the patch, slow bank voles furthermore invested more time in antipredatory vigilance than fast types (Mazza et al. 2019), decreasing their harvest rate curve (Brown and Kotler 2004) and indicating lower exploitation efficiency (but see Mella et al. 2015 for a contrasting pattern in brushtail possums, *Trichosurus vulpecula*). By relating time in a patch directly to patch exploitation across risk treatments, we revealed that a significant part of deviation of patch exploitation from the population-level harvest rate was due to among-individual differences. Thus, intrinsic differences in risk-taking appear to affect the perceived risk level and harvesting efficiency creating unequal payoffs among different foraging types, which are likely state dependent and might be part of an extended pace-of-life syndrome (POLS), where risk-taking behaviour is adaptively integrated into a suite of correlated behavioural, physiological and life-history traits (e.g. Dammhahn et al. 2018; Reale et al. 2007). Ultimately, under natural conditions, riskier foraging ought to come at the cost of reduced survival to maintain variation in foraging-under-risk styles, as e.g. in free-ranging wandering albatrosses, *Diomedea exulans*, in which different foraging styles had similar fitness over the long run (e.g. Patrick et al. 2017).

## Conclusions

Predation risk during foraging is a strong determinant of fitness for small ground-dwelling rodents (Norrdahl and Korpimaki 1998). By manipulating ground cover, we could modify perceived predation risk of singly foraging common voles and, thus, create an experimental landscape of fear (Brown 1999; Laundré et al. 2001; Brown and Kotler 2004). Supporting the classical patch use model (Brown 1988), risk during feeding was a strong predictor of foraging behaviour and of remaining resource landscapes. However, perceived risk in the matrix between food patches affected travelling costs. Increased travelling costs resulted in uneven resource exploitation across a uniform risk landscape and ultimately induced heterogeneity in resource distribution in remaining landscapes. Thus, the risk properties of the matrix contribute to patch exploitation patterns in risk-uniform landscapes. Unequal exploitation of resource patches likely has population-wide effects for foragers and will modify meta-population and community dynamics of the food species (e.g. McArthur et al. 2014; Toscano et al. 2016; Moran et al. 2017). Among-individual differences in foraging under risk ultimately lead to unequal payoffs. In our experimental landscapes liberated of direct predation effects, individuals foraging more gained higher total food resources overall. Additionally, a significant part of the variation in patch exploitation was due to among-individual differences suggesting that some foragers consistently over- or underexploited while exposing themselves to predation risk. Acknowledging intrinsic among-individual differences in quitting harvest rate and in resource exploitation efficiency, that are beyond variation in energeric state or sex,  in theoretical models of optimal foraging might allow more nuanced prediction on foraging under risk. Future studies should address whether among-individual differences in foraging in landscapes of resources and risks are adaptive. Furthermore, since individual variation in foraging behaviour can create variation in remaining resource landscapes, we need to address potential cascading effects at the resource level.

## Electronic supplementary material

Below is the link to the electronic supplementary material.

Supplementary file1 (DOCX 52 kb)

## Data Availability

The complete behavioural data set will be made available as supplemental material together with this article.
