# Supplementary material for: Among-individual differences in foraging modulate resource exploitation under perceived predation risk
Source: Oecologia. 2020 Nov 3;194(4):621–34. doi: 10.1007/s00442-020-04773-y (PMC7683444; doi:10.1007/s00442-020-04773-y)
Supplement: Supplementary file 1 — Supplementary file1 (DOCX 52 kb) [file 442_2020_4773_MOESM1_ESM.docx]

Electronic supplement to manuscript

**Among-individual differences in foraging modulate resource exploitation under perceived predation risk**

**Jana A. Eccard**^1^***^,^ Thilo Liesenjohann**^1^**, Melanie Dammhahn**^1^

^1^Animal Ecology, Institute for Biochemistry and Biology, University of Potsdam, Maulbeerallee 1, 14469 Potsdam, Germany

**Table ESM1:** Repeatability estimates adjusted for four different treatment levels. Shown are repeatability estimates (R) and their standard error (SE), lower and upper 95% confidence intervals and p-values from LR-tests. Significant repeatability is indicated with p-values in bold font.

| **Variable** | **R ± SE** | **Lower CI** | **Upper CI** | **p** |
| --- | --- | --- | --- | --- |
| Latency to emerge [sec] | 0.41 ± 0.10 | 0.16 | 0.63 | **<0.001** |
| Duration active [min] | 0.40 ± 0.11 | 0.15 | 0.63 | **<0.001** |
| Duration feeding [min] | 0.45 + 0.13 | 0.18 | 0.67 | **<0.001** |
| Tray changes | 0.46 + 0.08 | 0.13 | 0.71 | **0.018** |
| Absolute outtake [g] | 0.14 + 0.07 | 0.00 | 0.40 | 0.087 |
| Minimal GUD [g/l] | 0.22 + 0.15 | 0.00 | 0.47 | **0.017** |
| Evenness | 0.04 + 0.07 | 0.00 | 0.26 | 0.349 |

**Table ESM2:** Within-individual correlations between foraging duration and resource exploitation. Shown are posterior estimates and their 95% credibility estimates from bivariate Bayesian mixed effects models (see main text for details). Credibility estimates excluding zero are indicated in bold font.

| **Foraging variable** | **Exploitation variable** | **Estimate** | **Lower CI** | **Upper CI** |
| --- | --- | --- | --- | --- |
| Duration feeding | Minimal GUD [g/l] | **-0.243** | -0.475 | -0.003 |
| Duration feeding | Total food outtake [g] | **0.367** | 0.148 | 0.577 |
| Patch change frequency | Evenness | 0.011 | -0.282 | 0.298 |

**Table ESM3:** Behavioural data from 21 common voles (*Microtus arvalis*) in a foraging trial in simple landscapes (two food patches) with different risk treatments. Documentation of variables in ESM4

| **ID** | **Sex** | **Treat** | **RiskUniformity** | **Order** | **FeedRisk** | **TravRisk** | **Latency emerge** | **Nr of bouts** | **TrayChanges** | **Duration active** | **Duration left** | **Duration ight** | **min.1bout.left** | **min.1bout.right** | **GUD.left** | **GUD.right** |
| --- | --- | --- | --- | --- | --- | --- | --- | --- | --- | --- | --- | --- | --- | --- | --- | --- |
| T1 | m | 1 | SRU | 1 | Fcov | Wcov | 66.6 | 2 | 2 | 24.5 | 5.1 | 9.4 | 5.1 | 9.4 | 0.83 | 0.69 |
| T3 | m | 1 | SRU | 4 | Fcov | Wcov | 95.7 | 2 | 2 | 28.4 | 1.2 | 16.3 | 1.2 | 16.3 | 0.83 | 0.5 |
| T4 | f | 1 | SRU | 1 | Fcov | Wcov | 7.3 | 2 | 13 | 210.0 | 25.2 | 30.2 | 25.2 | 30.2 | 0.7 | 0.66 |
| T5 | f | 1 | SRU | 3 | Fcov | Wcov | 92.2 | 2 | 4 | 27.8 | 21.3 | 17.7 | 21.3 | 10.2 | 0.51 | 0.86 |
| T6 | m | 1 | SRU | 3 | Fcov | Wcov | 86.8 | 1 | 3 | 19.0 | 3.6 | 4.8 | 3.6 | 4.8 | 0.98 | 0.95 |
| T8 | m | 1 | SRU | 3 | Fcov | Wcov | 5.5 | 2 | 17 | 170.9 | 34.5 | 30.1 | 34.5 | 26.7 | 0.65 | 0.6 |
| T9 | m | 1 | SRU | 2 | Fcov | Wcov | 23.0 | 1 | 1 | 8.4 | 2.4 | 2.4 | 0.0 | 0.0 | 0.77 | 0.74 |
| T10 | f | 1 | SRU | 5 | Fcov | Wcov |  | 1 | 1 | 0.2 | 0.1 | 0.0 | 0.0 | 0.0 | 1 | 0.52 |
| T11 | m | 1 | SRU | 3 | Fcov | Wcov | 56.4 | 2 | 1 |  |  |  |  |  | 0.58 | 0.75 |
| T12 | f | 1 | SRU | 1 | Fcov | Wcov | 0.8 | 2 | 3 | 8.7 | 4.0 | 3.0 | 0.7 | 0.3 | 0.59 | 0.74 |
| T14 | m | 1 | SRU | 4 | Fcov | Wcov | 38.2 | 2 | 1 | 9.9 | 7.3 | 0.0 | 7.3 | 0.0 | 0.65 | 0.91 |
| T15 | f | 1 | SRU | 2 | Fcov | Wcov | 3.5 | 2 | 3 | 4.5 | 6.0 | 8.6 | 6.0 | 8.6 | 0.63 | 0.6 |
| T16 | m | 1 | SRU | 3 | Fcov | Wcov | 1.3 | 2 | 3 | 10.2 | 6.6 | 0.0 | 0.0 | 0.0 | 0.39 | 0.42 |
| T17 | f | 1 | SRU | 3 | Fcov | Wcov |  | 2 | 1 | 25.9 | 3.5 | 9.0 | 0.0 | 0.0 | 0.72 | 0.65 |
| T18 | m | 1 | SRU | 4 | Fcov | Wcov | 29.9 | 3 | 5 | 55.8 | 1.4 | 23.2 | 1.4 | 15.2 | 0.75 | 0.52 |
| T19 | m | 1 | SRU | 5 | Fcov | Wcov |  |  |  |  |  |  |  |  | 0.88 | 0.87 |
| T20 | f | 1 | SRU | 4 | Fcov | Wcov | 111.6 | 1 | 5 | 19.6 | 0.7 | 7.5 | 0.7 | 7.5 | 0.75 | 0.65 |
| T21 | m | 1 | SRU | 1 | Fcov | Wcov | 24.8 | 3 | 7 | 14.3 | 6.1 | 38.1 | 6.1 | 38.1 | 0.81 | 0.75 |
| T22 | f | 1 | SRU | 4 | Fcov | Wcov | 105.3 | 2 | 2 | 41.3 | 20.2 | 4.7 | 20.2 | 4.7 | 0.75 | 0.78 |
| T23 | f | 1 | SRU | 4 | Fcov | Wcov | 36 | 2 | 0 | 56.8 | 10.2 | 13.2 | 0.0 | 1.7 | 0.78 | 0.77 |
| T24 | m | 1 | SRU | 4 | Fcov | Wcov | 32 | 2 | 0 | 12.9 | 8.9 | 2.1 | 9.0 | 0.1 | 0.52 | 0.82 |
| T1 | m | 2 | RRU | 4 | Fop | Wop |  |  |  |  |  |  |  |  |  |  |
| T3 | m | 2 | RRU | 5 | Fop | Wop | 167 | 6 | 2 | 5.9 | 0.0 | 3.2 |  |  | 1 | 0.85 |
| T4 | f | 2 | RRU | 4 | Fop | Wop | 5 | 4 | 3 | 90.7 | 1.2 | 1.5 | 1.2 | 1.5 | 0.79 | 0.98 |
| T5 | f | 2 | RRU | 4 | Fop | Wop | 109 | 5 | 17 | 34.1 | 0.0 | 11.1 | 0.0 | 11.1 | 0.98 | 0.52 |
| T6 | m | 2 | RRU | 2 | Fop | Wop | 227 | 1 | 0 | 2.2 | 1.5 | 0.0 | 1.6 | 0.0 | 1 | 1 |
| T8 | m | 2 | RRU | 4 | Fop | Wop | 1 | 4 | 14 | 98.6 | 20.3 | 35.4 | 30.3 | 35.4 | 0.73 | 0.68 |
| T9 | m | 2 | RRU | 1 | Fop | Wop | 124 | 2 | 3 | 12.6 | 4.0 | 1.0 | 4.1 | 1.0 | 0.79 | 1 |
| T10 | f | 2 | RRU | 4 | Fop | Wop | 106 | 1 | 3 | 11.1 | 0.0 | 5.1 |  |  | 0.7 | 0.88 |
| T11 | m | 2 | RRU | 4 | Fop | Wop | 102 | 6 | 8 | 21.4 | 4.7 | 5.5 |  |  | 0.82 | 0.91 |
| T12 | f | 2 | RRU | 3 | Fop | Wop | 165 | 5 | 7 | 29.0 | 4.7 | 3.7 | 4.7 | 3.7 | 0.76 | 0.76 |
| T14 | m | 2 | RRU | 3 | Fop | Wop |  |  |  | 3.7 | 0.0 | 0.2 | 0.0 | 0.2 | 0.98 | 0.93 |
| T15 | f | 2 | RRU | 3 | Fop | Wop | 143 | 4 | 6 | 10.2 | 0.5 | 5.5 | 0.5 | 5.2 | 0.89 | 0.49 |
| T16 | m | 2 | RRU | 2 | Fop | Wop | 102 | 3 | 5 | 35.0 | 2.3 | 1.6 | 2.3 | 1.6 | 0.9 | 0.79 |
| T17 | f | 2 | RRU | 2 | Fop | Wop | 223 | 1 | 5 | 16.9 | 3.4 | 2.2 | 3.4 | 2.2 | 0.9 | 0.96 |
| T18 | m | 2 | RRU | 1 | Fop | Wop | 197 | 3 | 8 | 74.1 | 17.1 | 11.9 | 17.1 | 12.0 | 0.7 | 0.5 |
| T19 | m | 2 | RRU | 2 | Fop | Wop | 217 | 1 | 4 | 7.8 | 3.5 | 1.7 | 3.5 | 1.7 | 0.65 | 0.96 |
| T20 | f | 2 | RRU | 2 | Fop | Wop | 130 | 2 | 2 | 21.3 | 0.0 | 3.4 | 0.0 | 0.5 | 0.65 | 0.84 |
| T21 | m | 2 | RRU | 3 | Fop | Wop | 182 | 2 | 4 | 39.8 | 0.9 | 5.8 | 1.0 | 5.8 | 0.94 | 0.82 |
| T22 | f | 2 | RRU | 1 | Fop | Wop | 156 | 3 | 7 | 27.0 | 46.0 | 15.8 | 46.0 | 15.8 | 0.73 | 0.52 |
| T23 | f | 2 | RRU | 3 | Fop | Wop | 86 | 2 | 6 | 86.5 | 4.5 | 2.6 | 4.5 | 2.6 | 0.65 | 0.78 |
| T24 | m | 2 | RRU | 3 | Fop | Wop | 239 | 2 | 0 | 9.8 | 5.0 | 8.7 | 5.0 | 8.7 | 1 | 0.55 |
| T1 | m | 3 |  | 2 | Fcov | Wop | 181 | 2 | 1 | 14.7 | 3.9 | 7.6 | 4.0 | 7.6 | 0.85 | 0.75 |
| T3 | m | 3 |  | 1 | Fcov | Wop | 59 | 5 | 0 | 50.1 | 15.3 | 33.4 | 15.3 | 33.4 | 0.3 | 0.51 |
| T4 | f | 3 |  | 2 | Fcov | Wop | 0 | 2 | 15 | 111.6 | 61.4 | 50.2 | 61.5 | 50.2 | 0.5 | 0.87 |
| T5 | f | 3 |  | 2 | Fcov | Wop | 48 | 4 | 13 | 86.8 | 4.4 | 18.6 | 4.4 | 18.6 | 0.81 | 0.52 |
| T6 | m | 3 |  | 4 | Fcov | Wop | 212 | 2 | 2 |  |  |  |  |  |  |  |
| T8 | m | 3 |  | 1 | Fcov | Wop | 27 | 3 | 23 | 135.4 | 26.4 | 35.7 | 26.4 | 35.7 | 0.74 | 0.77 |
| T9 | m | 3 |  | 4 | Fcov | Wop | 120 | 3 | 3 | 5.3 | 0.7 | 1.8 | 0.7 | 1.8 | 0.94 | 0.89 |
| T10 | f | 3 |  | 1 | Fcov | Wop | 80 | 3 | 11 | 44.1 | 1.4 | 16.4 | 1.4 | 16.4 | 1 | 0.85 |
| T11 | m | 3 |  | 5 | Fcov | Wop | 140 | 1 | 1 | 15.2 | 4.3 | 7.4 | 4.3 | 7.4 | 0.69 | 0.81 |
| T12 | f | 3 |  | 4 | Fcov | Wop | 0 | 1 | 1 | 5.8 | 0.0 | 2.5 | 0.0 | 2.5 | 0.74 | 0.94 |
| T14 | m | 3 |  | 2 | Fcov | Wop | 91 | 3 | 3 | 28.1 | 1.1 | 2.8 | 1.1 | 2.8 | 0.88 | 0.96 |
| T15 | f | 3 |  | 1 | Fcov | Wop | 2 | 3 | 4 | 38.6 | 15.5 | 7.7 | 15.5 | 7.7 | 0.6 | 0.75 |
| T16 | m | 3 |  | 4 | Fcov | Wop | 4 | 1 | 7 | 27.6 | 1.4 | 1.0 | 1.4 | 1.0 | 0.91 | 0.74 |
| T17 | f | 3 |  | 5 | Fcov | Wop |  |  |  |  |  |  |  |  | 0.97 | 0.88 |
| T18 | m | 3 |  | 5 | Fcov | Wop | 38 | 3 | 6 | 52.2 | 4.6 | 7.7 | 4.6 | 7.7 | 0.9 | 0.75 |
| T19 | m | 3 |  | 1 | Fcov | Wop | 82 | 3 | 6 | 31.3 | 12.5 | 2.3 | 12.5 | 2.3 | 0.65 | 0.86 |
| T20 | f | 3 |  | 3 | Fcov | Wop | 171 | 1 | 1 | 17.4 | 3.9 | 10.3 | 3.9 | 10.3 | 0.84 | 0.57 |
| T21 | m | 3 |  | 4 | Fcov | Wop | 192 | 2 | 2 | 22.9 | 0.3 | 7.3 | 0.3 | 4.0 | 0.95 | 0.55 |
| T22 | f | 3 |  | 2 | Fcov | Wop | 8 | 3 | 6 | 63.7 | 19.9 | 26.2 | 19.9 | 26.2 | 0.71 | 0.88 |
| T23 | f | 3 |  | 1 | Fcov | Wop | 194 | 2 | 9 | 34.1 | 10.1 | 2.3 | 2.6 | 2.3 | 0.94 | 0.82 |
| T24 | m | 3 |  | 2 | Fcov | Wop | 69 | 2 | 3 | 31.7 | 1.4 | 11.4 | 1.4 | 11.4 | 0.68 | 0.32 |
| T1 | m | 4 |  | 3 | Fop | Wcov | 58 | 3 | 1 | 70.0 | 7.8 | 16.2 | 7.8 | 8.2 | 0.41 | 0.48 |
| T3 | m | 4 |  | 2 | Fop | Wcov | 93 | 4 | 1 | 27.7 | 5.8 | 10.8 | 5.8 | 10.8 | 0.76 | 0.55 |
| T4 | f | 4 |  | 5 | Fop | Wcov | 15 | 6 | 14 | 15.8 | 1.9 | 2.6 |  |  | 0.88 | 0.89 |
| T5 | f | 4 |  | 1 | Fop | Wcov | 43 | 3 | 12 | 66.3 | 2.0 | 145.0 | 2.0 | 145.0 | 0.8 | 0.72 |
| T6 | m | 4 |  | 5 | Fop | Wcov | 37 | 4 | 7 | 8.6 | 4.4 | 1.0 |  |  | 0.85 | 0.94 |
| T8 | m | 4 |  | 5 | Fop | Wcov | 40 | 3 | 25 | 56.2 | 23.4 | 15.8 |  |  | 0.72 | 0.76 |
| T9 | m | 4 |  | 3 | Fop | Wcov | 236 | 3 | 3 | 8.2 | 0.6 | 0.8 | 2.4 | 0.4 | 0.89 | 0.91 |
| T10 | f | 4 |  | 2 | Fop | Wcov | 1 | 2 | 6 | 53.8 | 0.2 | 3.7 | 0.2 | 3.7 | 0.95 | 0.95 |
| T11 | m | 4 |  | 2 | Fop | Wcov | 14 | 5 | 9 | 22.0 | 9.3 | 0.9 |  |  | 0.95 | 0.95 |
| T12 | f | 4 |  | 5 | Fop | Wcov | 2 | 2 | 3 | 6.9 | 0.5 | 0.0 | 0.5 | 0.0 | 0.6 | 0.9 |
| T14 | m | 4 |  | 1 | Fop | Wcov | 215 | 3 | 2 | 18.5 | 0.3 | 0.3 | 0.4 | 0.3 | 0.92 | 0.91 |
| T15 | f | 4 |  | 5 | Fop | Wcov | 24 | 4 | 6 | 2.8 | 0.0 | 4.4 | 0.0 | 4.4 | 0.89 | 0.92 |
| T16 | m | 4 |  | 5 | Fop | Wcov |  |  |  |  |  |  |  |  | 0.81 | 0.69 |
| T17 | f | 4 |  | 1 | Fop | Wcov | 15 | 2 | 13 | 50.8 | 6.2 | 4.0 | 6.2 | 4.0 | 0.55 | 0.68 |
| T18 | m | 4 |  | 3 | Fop | Wcov | 273 | 1 | 1 | 19.5 | 2.7 | 1.7 | 2.7 | 1.7 | 0.79 | 0.82 |
| T19 | m | 4 |  | 4 | Fop | Wcov | 191 | 1 | 1 | 6.3 | 0.1 | 0.9 | 0.1 | 0.9 | 0.91 | 0.91 |
| T20 | f | 4 |  | 5 | Fop | Wcov | 121 | 0 | 2 | 30.8 | 2.4 | 14.4 | 2.3 | 14.5 | 0.84 | 0.82 |
| T21 | m | 4 |  | 5 | Fop | Wcov | 25 | 2 | 7 | 42.5 | 7.3 | 6.1 | 7.3 | 6.1 | 0.65 | 0.68 |
| T22 | f | 4 |  | 3 | Fop | Wcov | 91 | 2 | 4 | 51.9 | 13.1 | 9.2 | 13.1 | 9.2 | 0.6 | 0.62 |
| T23 | f | 4 |  | 5 | Fop | Wcov | 110 | 2 | 1 | 56.1 | 4.2 | 3.3 | 4.2 | 3.3 | 0.88 | 0.82 |
| T24 | m | 4 |  | 1 | Fop | Wcov | 101 | 2 | 4 | 24.4 | 5.0 | 5.3 | 5.0 | 5.3 | 0.59 | 0.71 |
| T1 | m | 5 | RH | 5 |  |  | 40 | 3 | 1 |  | 9.0 |  |  |  | 0.42 | 1 |
| T3 | m | 5 | RH | 3 |  |  | 74 | 2 | 1 | 45.2 | 33.1 | 0.1 | 33.1 | 0.1 | 0.21 | 1 |
| T4 | f | 5 | RH | 3 |  |  | 5 | 3 | 7 | 150.5 | 22.5 | 77.5 | 22.5 | 77.5 | 0.86 | 0.78 |
| T5 | f | 5 | RH | 5 |  |  | 42 | 3 | 3 | 16.2 | 0.8 | 12.0 |  |  | 0.97 | 0.31 |
| T6 | m | 5 | RH | 1 |  |  | 115 | 1 | 1 | 17.8 | 8.2 | 1.6 | 8.2 | 1.6 | 0.98 | 0.54 |
| T8 | m | 5 | RH | 2 |  |  | 56 | 3 | 19 | 146.9 | 47.8 | 36.2 | 47.9 | 36.2 | 0.81 | 0.42 |
| T9 | m | 5 | RH | 5 |  |  | 116 | 2 | 0 | 16.1 | 10.0 | 0.0 | 10.0 | 0.0 | 0.49 | 1 |
| T10 | f | 5 | RH | 3 |  |  |  |  |  |  |  |  |  |  | 0.9 | 1 |
| T11 | m | 5 | RH | 1 |  |  | 223 | 1 | 0 | 19.6 | 0.0 | 14.1 |  |  | 0.91 | 0.41 |
| T12 | f | 5 | RH | 2 |  |  | 131 | 2 | 0 | 0.4 | 0.0 | 0.0 |  |  | 1 | 0.79 |
| T14 | m | 5 | RH | 5 |  |  |  |  |  |  |  |  |  |  | 0.98 | 0.41 |
| T15 | f | 5 | RH | 4 |  |  | 126 | 1 | 1 | 12.7 | 6.5 | 0.1 | 6.5 | 0.1 | 0.44 | 0.79 |
| T16 | m | 5 | RH | 1 |  |  | 92 | 3 | 7 | 38.2 | 3.5 | 0.6 | 3.5 | 0.6 | 0.79 | 0.97 |
| T17 | f | 5 | RH | 4 |  |  | 84 | 3 | 1 | 25.3 | 6.2 | 4.3 | 6.2 | 4.3 | 0.72 | 0.83 |
| T18 | m | 5 | RH | 2 |  |  | 64 | 3 | 3 | 55.9 | 17.2 | 9.0 |  |  | 1 | 0.89 |
| T19 | m | 5 | RH | 3 |  |  | 227 | 2 | 2 | 6.3 | 0.6 | 1.3 | 0.6 | 1.3 | 0.98 | 0.88 |
| T20 | f | 5 | RH | 1 |  |  | 128 | 2 | 2 | 41.0 | 1.9 | 0.8 | 1.9 | 0.8 | 0.75 | 0.97 |
| T21 | m | 5 | RH | 2 |  |  | 161 | 2 | 6 | 39.9 | 5.8 | 13.3 | 11.4 | 2.5 | 0.84 | 0.93 |
| T22 | f | 5 | RH | 5 |  |  | 117 | 2 | 2 | 51.5 | 31.2 | 0.1 | 31.2 | 0.1 | 0.68 | 0.96 |
| T23 | f | 5 | RH | 2 |  |  | 86 | 2 | 3 | 54.2 | 1.9 | 122.3 | 2.0 | 122.3 | 0.97 | 0.81 |
| T24 | m | 5 | RH | 5 |  |  | 92 | 2 | 3 | 23.7 | 2.5 | 10.7 | 2.5 | 10.7 | 0.93 | 0.72 |

**Table ESM4**: Documentation of treatments and behavioural variables from ESM3

**Animal**

ID Animal ID (for body weight and arena see also subject table ESM5)

Sex m:male, f: female

**Experimental Treatment**

Treat Treatments numbered

Risk Uniformity Name of treatment in Eccard and Liesenjohann 2014, SRU: safe, risk-uniform; RRU: risky, risk-uniform; RH: risk heterogeneity. Note that treatment RH was not analysed in the current paper

Order Order of treatments within animal

FeedRisk Fcov: foot patches covered (safe), Fop: Food patches open (risky)

TravRisk Wcov: ground between trays covered (safe), Wop: open (risky)

**Behavioural Data (Video analysis)**

Latency emerge Latency to commence activity after researcher left (minutes)

Nr of bouts Number of activity bouts separated by >30min inactivity

TrayChanges Number of changes from one tray to the other

Duration active Activity in the arena (minutes)

Duration.left Time in left tray (minutes)

Duration.right Time in right tray (minutes)

min.1bout.left Time in left tray first activity bout (minutes)

min.1bout.right Time in right tray first activity bout (minutes)

**Foraging Landscape**

GUD.left Giving up density of millet seeds (g / liter) in left tray

GUD.right Giving up density of millet seeds (g / liter) in right tray

**Reference:**

Eccard JA, Liesenjohann T (2014) The Importance of Predation Risk and Missed Opportunity Costs for Context-Dependent Foraging Patterns. PLoS ONE 9:e94107. doi: 10.1371/journal.pone.0094107

**Table ESM5**: Information on subjects (vole individuals) from Table ESM4. BW1: body mass (in g) measured when introducing animal to arena, BW2: body mass (in g) measured after 3-day habituation. Round: group of 8 animals tested simultaneously, Arena: numbered arenas in four different aviaries (of two arenas each).

| **ID** | **Sex** | **BW1** | **BW2** | **Round** | **Arena** | **Aviary** |
| --- | --- | --- | --- | --- | --- | --- |
| T1 | 2 | 23 | 20 | 1 | 1 | a |
| T2 | 1 | 18 | 16 | 1 | 2 | a |
| T3 | 2 | 24 | 18 | 1 | 3 | b |
| T4 | 1 | 24 | 18 | 1 | 4 | b |
| T5 | 1 | 23 | 18,5 | 1 | 5 | c |
| T6 | 2 | 22 | 18 | 1 | 6 | c |
| T7 | 1 | 20 | 19 | 1 | 7 | d |
| T8 | 2 | 20 | 16 | 1 | 8 | d |
| T9 | 2 | 32 | 28 | 2 | 1 | a |
| T10 | 1 | 26 | 24 | 2 | 2 | a |
| T11 | 2 | 30 | 27 | 2 | 3 | b |
| T12 | 1 | 28 | 26 | 2 | 4 | b |
| T13 | 1 | 29 | 27 | 2 | 5 | c |
| T14 | 2 | 30 | 28 | 2 | 6 | c |
| T15 | 1 | 28 | 25 | 2 | 7 | d |
| T16 | 2 | 30 | 29 | 2 | 8 | d |
| T17 | 1 | 25 | 23 | 3 | 1 | a |
| T18 | 2 | 30 | 28 | 3 | 2 | a |
| T19 | 2 | 30 | 27 | 3 | 3 | b |
| T20 | 1 | 25 | 23 | 3 | 4 | b |
| T21 | 2 | 28 | 26 | 3 | 5 | c |
| T22 | 1 | 24 | 22 | 3 | 6 | c |
| T23 | 1 | 25 | 22 | 3 | 7 | d |
| T24 | 2 | 24 | 23 | 3 | 8 | d |
|  |  |  |  |  |  |  |
